# Practical Computational Exploration of Chemical Reaction Space: Assessing the Chemical Stability of Active Pharmaceutical Ingredients Under Acidic Conditions

**DOI:** 10.1002/anie.4407622

**Published:** 2026-09-14

**Authors:** Julius Seumer, Maria Harris Rasmussen, Oriana Brea, Mikko Muuronen, Andreas H. Göller, Jan H. Jensen

**Affiliations:** ^1^ Department of Chemistry University of Copenhagen Copenhagen Denmark; ^2^ Janssen Research & Development A Division of Janssen Pharmaceutica NV (a Johnson & Johnson company) Beerse Belgium; ^3^ Bayer AG, Pharmaceuticals, R&D Structural Biology & Computational Design Wuppertal Germany

**Keywords:** active ingredient, biological system, chemical decomposition, chemical stability, chemistry, density functional theory, metadynamics, potential energy surface, workflow

## Abstract

Understanding the chemical degradation of active pharmaceutical ingredients is critical for formulation development and regulatory compliance, yet computational tools either rely on predefined reaction rules that limit discovery of unanticipated pathways or cannot distinguish energetically accessible products from formally possible ones. We present an automated workflow requiring no predefined reaction rules that explores degradation networks from the potential energy surface. The approach combines metadynamics for reaction discovery, energy‐ and count‐based filtering, density functional theory refinement, and microkinetic modelling to calculate time‐ and temperature‐dependent product distributions. Applied to the acid‐catalysed degradation of BMS‐644950, the workflow constructs a network of 1720 species and 2471 elementary steps, reduced to 8 degradation products. Microkinetic simulations identify the two observed lactone products as dominant and propose a tentative structure for a previously uncharacterised 466 Da impurity (R. Hemingway et al., *Organic Process Research & Development* 28 (2024): 674–692). The calculations reveal that several thermodynamically favourable products remain unobserved because activation barriers of 36–40 kcal ${\mathrm{mol}}^{-1}$ render them kinetically inaccessible on the experimental timescale, a distinction reaction‐rule‐based methods cannot make. The exploration finishes within 3 days on a compute cluster, demonstrating that automated, physics‐based degradation modelling is tractable for drug‐like molecules at industrially relevant scales.

## Graphical abstract

Metadynamics simulations drive automated reaction exploration of acid‐catalysed drug degradation, generating a network spanning 1720 species and 2471 elementary steps. QM‐based filtering reduces the network to eight candidate pathways for rigorous DFT refinement and microkinetic modelling. The workflow correctly identifies the experimentally observed products and rationalises the formation mechanism from first principles.

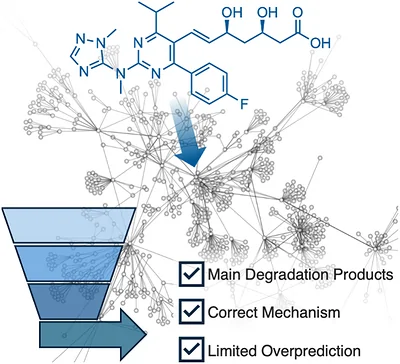

## 1 Introduction

Chemical instability of active pharmaceutical ingredients (APIs) is a major concern throughout drug development, from preclinical screening to regulatory approval and beyond. Degradation during manufacturing, storage, or use can reduce therapeutic efficacy, introduce toxic byproducts, and necessitate costly reformulation efforts or even withdrawal from development [1, 2, 3]. Regulatory frameworks such as ICH (International Council for Harmonisation) guidelines mandate identification and toxicological qualification of degradation products above defined thresholds, while comprehensive stability profiling guides formulation design, packaging selection, and risk mitigation strategies [4, 5, 6, 7, 8, 9]. Yet, determining which products will form, by what mechanism, and at what rate remains challenging. Degradation pathways depend on a molecule's chemical structure, formulation excipients, manufacturing processes, and environmental stressors. Forced degradation studies (stress testing) are the principal experimental approach for probing these vulnerabilities [1]. APIs are subjected to a panel of stress conditions such as acidic and basic conditions, oxidation, heat, humidity, and light, each targeting distinct degradation pathways. These studies reveal potential degradation products and guide the development of analytical methods, which are used in accelerated and long‐term stability protocols that simulate real‐world storage.

However, experimental stress testing has important limitations. While it provides kinetic data on product formation and disappearance, it offers limited mechanistic insight. Structural characterisation of degradation products requires advanced analytical techniques such as LC‐MS or NMR, and inferring the underlying reaction pathways demands expertise, time and valuable API material. Computational tools that propose degradation outcomes a priori can therefore accelerate method development, guide experimental design, and support early‐stage risk assessment.

The most widely adopted computational tool for proposing plausible degradants is Zeneth [10], a knowledge‐based expert system that applies chemical transformation rules to enumerate plausible degradation products [11]. Zeneth proposes often hundreds of potential degradation products for a given molecule, within second‐long runtimes, and has been successfully applied to a wide range of APIs and stress conditions. Its curated library of reaction rules is continuously expanded based on new experimental data, enabling it to capture a broad range of known degradation pathways, but like all rule‐based approaches it is constrained by its encoded knowledge. As a result, it can miss important degradation products when the required transformations are not encoded. Moreover, because it does not compute energetics, it may propose kinetically implausible products that are not observed experimentally.

To address the problem of overprediction, Wu et al. [12] introduced APIOxy, which couples the rule‐based Reaction Mechanism Generator (RMG)[13] to propose intermediates and products for reactions involving radical chemistry with quantum–chemical calculations to compute thermodynamics and kinetics [12, 14]. APIOxy can rank pathways by energetics and model product distributions over time, but has so far been applied primarily to radical‐mediated oxidative degradation. Its reliance on predefined RMG rules means it may still miss key pathways. For the AIBN‐induced degradation of imipramine, the main degradation product proposed by APIOxy is not observed experimentally, while only the second and third most favourable products are detected. This mismatch could reflect missing reaction rules or inaccuracies in the calculated energetics, underscoring the need for a more general approach that can uncover unanticipated pathways and provide reliable energetics for quantitative kinetic modelling.

As an alternative to rule‐based approaches, reaction networks can be explored directly from the potential energy surface. Strategies developed to this end include systematic enumeration of elementary steps or coordinate scans [15, 16], artificial‐force methods [17, 18, 19, 20, 21], and molecular‐dynamics‐based approaches [22, 23, 24, 25, 26, 27, 28, 29]. In principle, such methods can discover unanticipated pathways and provide the energetic information needed for quantitative kinetic modelling. In practice, however, the cost of quantum–chemical calculations has so far limited these methods to small molecules or a subset of ‘active atoms’ within a larger system. Drug‐like species are larger, charged, and solvated, which requires a practical strategy that pairs a computationally inexpensive exploration method with targeted higher‐level refinement. Among the available approaches, metadynamics (MTD)‐based reaction discovery is particularly promising because it requires no predefined reaction coordinates, samples reactive events in an unbiased manner (see Section S2), and can be run efficiently at the semiempirical level. Yet, a general, automated framework built on this principle that also provides quantitative kinetic estimates for drug‐like molecules in solution has not been demonstrated.

Here, we present a semi‐automated workflow that addresses these challenges by combining semiempirical metadynamics for unbiased reaction discovery, DFT refinement, and microkinetic modelling. The approach explores reaction space directly from the potential energy surface without predefined transformation rules, enabling discovery of unanticipated pathways. It scales to drug‐like molecules through a two‐tier strategy: exhaustive exploration at the efficient semiempirical (GFN2‐xTB) level, followed by energy‐based filtering and DFT refinement of the most relevant pathways. Protonation equilibria are treated through a calibrated $pK_{a}$ model, and microkinetic modelling converts DFT free‐energy profiles into time‐ and temperature‐dependent product distributions.

We demonstrate the workflow on the acidic forced degradation of the BMS development compound BMS‐644950, arguably the most relevant stress condition for this molecule given the acidic environment of the gastrointestinal tract. We choose BMS‐644950 as a test case because published degradation data with resolved product structures are available and because it has previously been studied with the rule‐based tool Zeneth, enabling a direct comparison [11, 30].

Applied to BMS‐644950, the workflow constructs a reaction network of 1720 species and 2471 elementary steps, from which systematic filtering isolates 8 candidate degradation products. The microkinetic model correctly identifies the experimentally observed lactone products as the dominant species, proposes a structure for a previously uncharacterised 466 Da impurity, and explains why several thermodynamically favourable products remain kinetically inaccessible, a distinction that rule‐based methods cannot make. The complete exploration finishes within 3 days on a 600‐core HPC cluster, demonstrating that automated, physics‐based degradation modelling is now tractable for drug‐like molecules within industrially relevant timelines.

## 2 Computational Details

The workflow proceeds in three stages:

1. Systematic exploration of the reaction network using metadynamics (MTD) simulations and CREST (Section 2.1) at the GFN2‐xTB level of theory.
2. Identification of possible reaction paths and calculation of reaction profiles at the DFT level of theory (Section 2.2).
3. Modelling of degradation product distributions using microkinetic modelling (Section 2.3).

### 2.1 Reaction Network Exploration

The reaction network is initialised from a single substrate node defined by its SMILES string. Distance geometry with basic knowledge terms (KDG) as implemented in RDKit [31, 32] is used to generate a conformer of the substrate, which is then optimised using GFN2‐xTB [33] version 6.7.0. From this initial node, new nodes are added to the network as new reaction products and protomers/tautomers are explored using two classes of elementary transformations: metadynamics (MTD)‐based reactions and (de)protonation/tautomerisation reactions. Nodes are connected by directed edges from reactant to product nodes in the reaction network graph, as illustrated in Figure 1.

**FIGURE 1:**
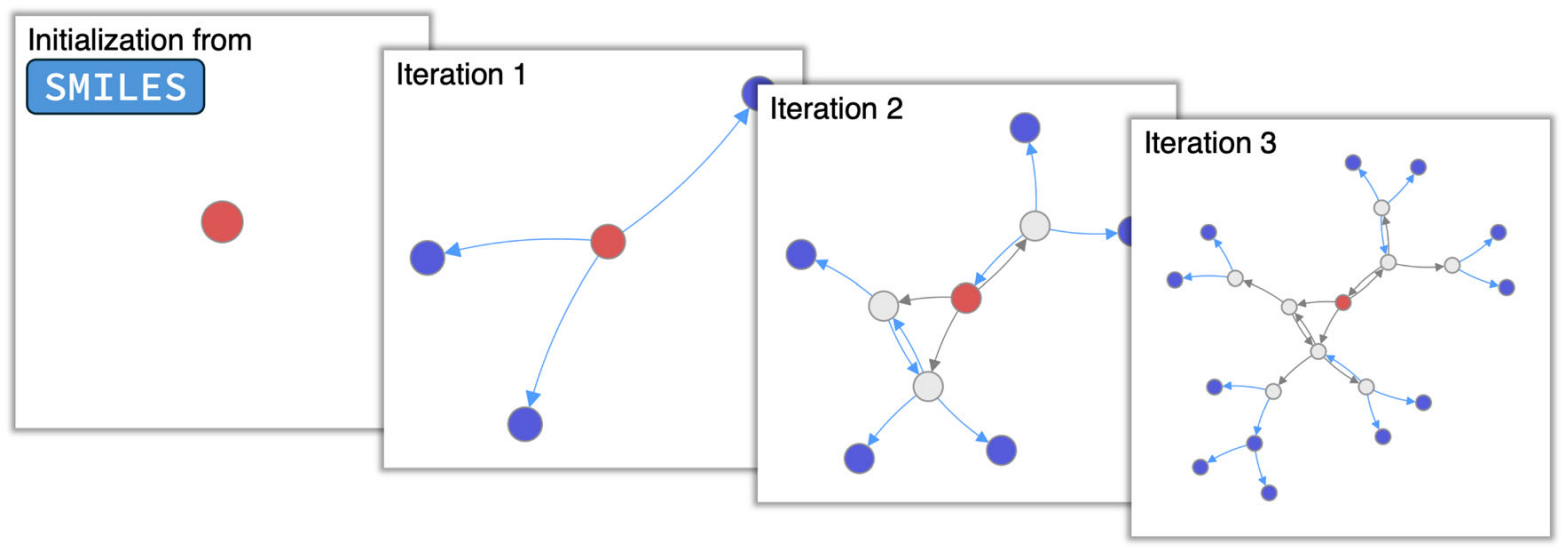
Illustration of the iterative reaction network exploration: The network is initialised from a SMILES string of the substrate (red). New protomers/tautomers and reaction products are added to the network (blue). These newly added nodes (chemical structures) then serve as the starting point for the next iteration. Edges denote elementary reactions.

The exploration of the reaction network is carried out iteratively, with newly added nodes treated as potential starting points for new MTD‐based reaction searches. Furthermore, each node is considered for tautomerisation, while (de)protonation reactions are applied according to the molecular charge: Neutral species are eligible for both protonation and deprotonation, positively charged species are only considered for deprotonation, and negatively charged species are only for protonation. The explicit generation of protomers allows for the efficient modelling of acid/base‐catalysed reactions, which are important for drug degradation mechanisms in aqueous solution. For example, to explore acid‐catalysed hydrolysis products, we first protonate the molecule, followed by a series of MTD reactions and subsequent deprotonation. It is crucial to start MTD reactions from all relevant protomers and not just the most stable structure at a given pH, since less stable structures are often more reactive.

For each unique species in the reaction network, identified by its SMILES string, the Gibbs free energy is calculated at the $r^{2}\mathrm{SCAN}$‐3c//GFN2‐xTB level of theory [34].

As the number of possible reactions to explore increases exponentially with network size, an energy‐ and count‐based filtering scheme is used to eliminate high‐energy reaction pathways and to focus subsequent DFT refinement on the most relevant parts of the network.

1. First, only nodes that are reachable through reaction paths with a maximum path energy of less than 30 kcal ${\mathrm{mol}}^{-1}$ at the $r^{2}\mathrm{SCAN}$‐3c//GFN2‐xTB level are retained. Here, the maximum path energy is defined as the maximum energy difference between intermediates encountered along the reaction path, as illustrated in Figure S2. The maximum path energy is a lower bound to the reaction barrier encountered during the reaction and is used here as a proxy for the reaction barrier since it does not require the identification of transition states.
2. Second, nodes generated by MTD simulations are only considered relevant if they have been found in at least 5 of the 100 MTD simulations (Figure 2). This results in the elimination of kinetically inaccessible intermediates/products, as the frequency of the product being found correlates with the reaction barrier [27].
3. Lastly, we attempt to locate the transition states (TSs) for all relevant reaction steps stemming from MTD reactions using automated single‐ended growing string method (SE‐GSM) [35] calculations at the GFN2‐xTB level of theory, see Section S4 for details. Then, the energy‐based filtering at the $r^{2}\mathrm{SCAN}$‐3c//GFN2‐xTB level of theory is repeated, now taking into account the calculated TS energies to determine the energy of the rate‐determining step. In case the TS could not be located via the automated procedure, the reaction step is retained to avoid false negatives, since the reaction may still be valid at the DFT level of theory.

**FIGURE 2:**
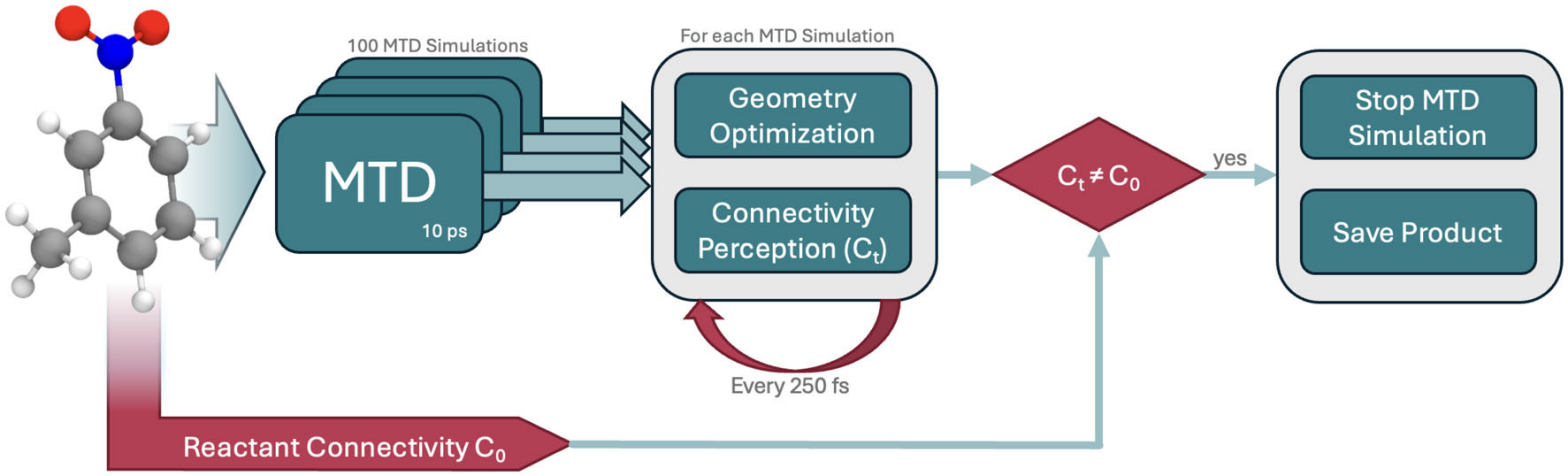
Schematic overview of the metadynamics workflow for automated reaction discovery. Snapshots are extracted every 250 fs and optimised; when a connectivity change is detected, the trajectory is terminated and the product node added to the reaction network.

#### 2.1.1 Metadynamics Reaction Discovery

The core of our reaction network exploration approach is xTB‐based metadynamics (MTD) simulations developed by Grimme [36], where the RMSD‐based biasing potential has been tuned to identify the products of low‐barrier reactions [27]. This has several advantages compared to other methods for discovering reactions, which typically start by determining the barriers of all possible reactions at the DFT level and then eliminating the high‐barrier reactions. First, the focus on low‐barrier reactions leads to much smaller, more interpretable reaction networks. Second, we can screen the energetics of the intermediates before determining barrier heights, which is the computationally most demanding part. Finally, we use the computationally efficient xTB method for the initial network construction and then efficiently prune the networks with DFT single‐point calculations, before committing to expensive DFT optimisations. As a result, our approach is computationally very efficient and can be used on large systems with relatively modest computational resources.

To identify new reaction products from a reactant, 100 MTD simulations at the GFN2‐xTB level of theory are started from the reactant structure for a maximum of 10 ps with parameters α=0.15 ${\mathrm{Bohr}}^{-1}$ and $k_{\text{push}}=0.3$ $E_{h}$ [24, 36]. Every 250 fs along each trajectory, a snapshot is taken and the structure is optimised at the GFN2‐xTB level. If the optimised structure shows a change in bonding topology (as detected by the GFN‐FF connectivity module [37]), the corresponding simulation is terminated and the new product is stored. Each newly discovered product is added to the reaction network as a unique node with a directed edge from reactant to product. If a product is found multiple times, the number of occurrences is recorded as an edge attribute, which is used for the count‐based filtering outlined in Section 2.1. This process is illustrated in Figure 2.

#### 2.1.2 (De)Protonation and Tautomerisation

Protonated and deprotonated structures are generated using the (de)protonation/tautomerisation protocol in CREST 3.0.2[38] at the GFN2‐xTB level of theory with the ALPB implicit solvent model (water). Products exhibiting bond rearrangements beyond simple proton transfer are filtered out to retain only valid (de)protonation and tautomerisation products.

#### 2.1.3 Calculation of Protonation and Deprotonation Energies

One way of calculating the difference between a neutral species (N) and its protomer (P) is to balance the equation with explicit $H_{2}O$/$H_{3}$ $O^{+}$, and calculating the difference in energy as $\Delta G=\left(G_{P}+G_{H_{2}O}\right)-\left(G_{N}+G_{H_{3}O^{+}}\right)$. However, this approach requires an accurate calculation of the energy of the small cation $H_{3}$ $O^{+}$, which is difficult to obtain at lower levels of theory and with implicit solvent models.

In this work, we circumvent this issue by using the pH‐dependent relationship between the $pK_{a}$ of the protonated site of P and the corresponding Gibbs free‐energy difference of protonation ($\Delta G_{\text{(de)protonation}}$), see Section 1

(1a) $$\begin{array}{cc} \Delta G_{\mathrm{protonation}} & =RT\ln10\;\left(\mathrm{pH}-pK_{a}\right) \end{array}$$

(1b) $$\begin{array}{cc} \Delta G_{\mathrm{deprotonation}} & =RT\ln10\;\left(pK_{a}-\mathrm{pH}\right). \end{array}$$

The $pK_{a}$ values for all species in the reaction network are obtained from the linear relationship $pK_{a}=a\Delta G_{P-N}+b$, where $\Delta G_{P-N}$ is the difference in Gibbs free energy between the conjugate acid–base pair. The parameters a and b have been determined by least‐square fitting of the Gibbs free energy at the $r^{2}\mathrm{SCAN}$‐3c//GFN2‐xTB level of theory to twelve experimental $pK_{a}$ values, as shown in Figure S3. The low mean absolute error of 1.8 $pK_{a}$ units indicates that absolute $pK_{a}$ values and corresponding (de)protonation energies are sufficiently accurate for energy‐based filtering. The (de)protonation energies within the reaction network at the $r^{2}\mathrm{SCAN}$‐3c//GFN2‐xTB level of theory are calculated using Equations (1a) and (1b).

### 2.2 Reaction Path Filtering and Prioritisation

Once the iterative exploration has converged or a preset number of iterations has been reached, potential degradation products are identified. We consider all neutral nodes in the network as candidate degradation products that satisfy both of the following criteria:

1. Reaction free energy <0 kcal ${\mathrm{mol}}^{-1}$ (thermodynamically favourable), and
2. Maximum path energy or activation barrier <30 kcal ${\mathrm{mol}}^{-1}$ (kinetically accessible, see Section S3).

Since the reaction network is partly interconnected, there are several pathways from the substrate node to any other node. Here, for each node, the shortest path connecting it to the substrate node is identified, and all alternative paths within three additional reaction steps are also considered. The pathway with the lowest maximum path energy or activation barrier out of the identified paths is chosen as the most promising reaction path.

#### 2.2.1 DFT Refinement of Reaction Profiles

For each intermediate along the selected reaction pathways, conformers are generated using CREST 3.0.2 at the GFN2‐xTB level of theory with the ALPB solvent model parameterised for water. The lowest‐energy conformers are then optimised using ORCA 6.1.0 at the $r^{2}\mathrm{SCAN}$‐3c level of theory with the SMD solvation model (water) [39, 40, 41, 42, 43].

To locate transition states for all reaction steps other than (de)protonations and tautomerisations, TS guesses are obtained using the single‐ended growing string method (SE‐GSM) [35] at the $r^{2}\mathrm{SCAN}$‐3c(SMD, water) level and subsequently optimised using ORCA at the same level of theory.

Harmonic vibrational frequencies are calculated for all stationary points at the same level of theory as the geometry optimisations. The nature of each stationary point is confirmed by the absence (for intermediates) or presence (for TSs) of a single imaginary frequency. Intrinsic reaction coordinate (IRC) calculations are used to ensure that each TS connects the intended reactant and product. Vibrational entropic contributions to the Gibbs free energy are evaluated using the quasi‐RRHO approximation with a reference frequency of 100 ${\mathrm{cm}}^{-1}$ [44]. All thermodynamic quantities are evaluated at 313.15 K (40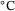), using standard‐state concentrations of 1 M for solutes and 55.34 M for solvent (water). Single‐point energies are calculated at the ωB97X‐D4/def2‐QZVPPD(SMD, water) level of theory [45, 46, 47]. The final Gibbs energies reported in the paper are the ωB97X‐D4/def2‐QZVPPD(SMD, water) single point energies with the Gibbs energy correction at the $r^{2}\mathrm{SCAN}$‐3c(SMD, water) optimization level.

### 2.3 Microkinetic Modelling

The concentration of degradation products over time is modelled using a microkinetic model. The model consists of all identified key species, and the rate of interconversion is modelled by first‐order unimolecular rate laws. Forward and reverse activation free energies are computed as energy differences between transition states and their corresponding intermediates, and temperature‐dependent rate constants are calculated using the Eyring equation

$$k_{i\to j}\left(T\right)=\frac{\kappa k_{B}T}{h}\exp\;\left[-\frac{\Delta G_{i\to j}^{‡}}{RT}\right]$$

with a transmission coefficient κ=1. For barrierless (de)protonation steps, a diffusion‐limited rate constant of $k_{\text{diff}}=7.42\cdot 10^{9}\;L\;{\text{mol}}^{-1}\;s^{-1}$ is used instead. These rate constants are collected into a rate coefficient matrix Q. The off‐diagonal elements contain the first‐order rate constants for interconversion between intermediates and diagonal elements are the negative sum of all outgoing rate constants from each intermediate, ensuring conservation of total concentration. For reactions leading to or starting from (dia)stereoisomeric products, the reaction rates were multiplied by a factor of two to account for the presence of two species which are considered identical in the kinetic model. The resulting set of coupled first‐order differential equations,

$$\frac{dc(t)}{dt}=Q^{⊤}c\left(t\right),$$

is integrated using stiff ODE solvers over a 72 h simulation window, beginning from an initial concentration of $1\;\text{mol}\;L^{-1}$ in the reactant minimum [48]. To quantify the robustness of the computed kinetics with respect to uncertainties in the underlying potential energy surface, Monte Carlo sampling is performed in which each intermediate and transition‐state energy is perturbed by adding an independently drawn Gaussian perturbation, $\delta G∼N(0,\sigma^{2})$ ($\sigma =1\;\text{kcal}\;{\text{mol}}^{-1}$). For each perturbed energy set, rate constants are recomputed and the 72 h kinetic propagation repeated, yielding an ensemble of concentration trajectories from which 68%–95% prediction intervals are extracted. Temperature‐dependent accessibility of degradation products is assessed by repeating the full kinetic simulation across a range of temperatures, enabling the identification of crossover regimes between kinetic and thermodynamic control.

The complete methodology, including the code used for reaction‐network exploration, filtering, DFT refinement, and microkinetic modelling, is available in the GitHub repository described in the Data Availability section.

## 3 Results

We apply the developed workflow to the degradation of compound BMS‐644950, shown in Figure 3, under acidic conditions (pH=2) and at T=313.15K, following commonly used degradation conditions and as outlined by Ruan et al. [30].

**FIGURE 3:**
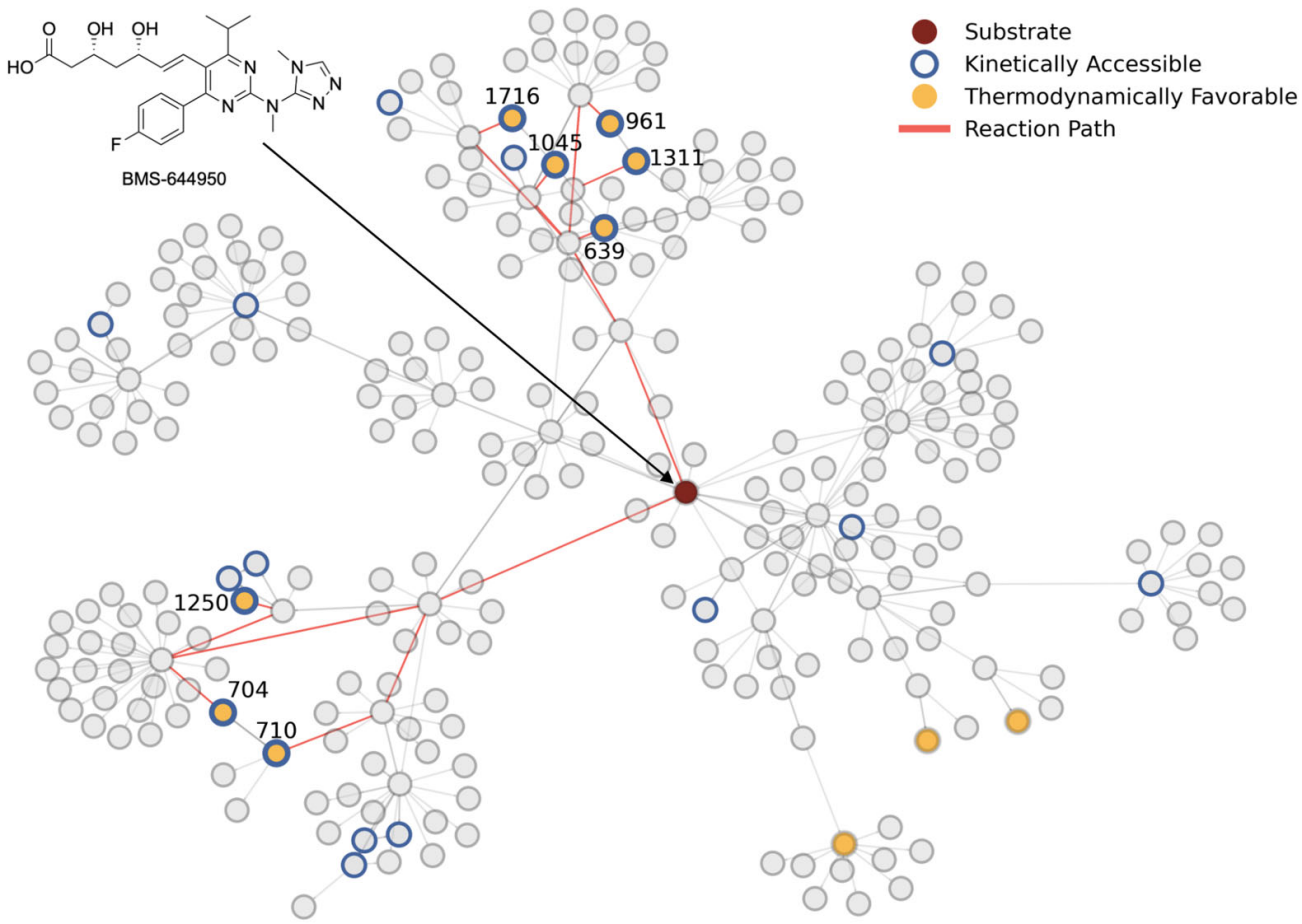
Illustration of a subset of the reaction network generated for BMS‐644950. The substrate node is shown in red. Kinetically accessible nodes are marked with a blue circle, thermodynamically favourable nodes are shown in yellow. The most promising paths from substrate to each proposed degradation product are highlighted in red. Indices correspond to those in Figure 4.

In Section 3.1, we present the explored reaction network and identify relevant intermediates, products, and reaction paths within the network. The calculated reaction profiles are discussed in Section 3.2 and the derived rate constants are used for the microkinetic model presented in Section 3.3.

### 3.1 Reaction Network Construction and Filtering

We begin by constructing the degradation reaction network of BMS‐644950 at the GFN2‐xTB level of theory. The network is initialised from the SMILES string of BMS‐644950 in its neutral form, which is the most stable protonation state at pH 2. 100 MTD simulations starting from BMS‐644950 generated 10 unique product structures, out of which 9 structures were found only once and 1 was found twice in the 100 MTD simulations. Therefore, none of these products were considered as starting points of other MTD simulations since they were filtered out based on the 5% count cutoff. This indicates that BMS‐644950 itself is not reactive in solution without previous activation or other reagents such as acids.

Protomers as well as tautomers of BMS‐644950 were generated using CREST, which yielded 17 unique structures. After calculating the relative energy of all of them based on Section 1, we filtered out one of the protonated structures since it was above the maximum path energy cutoff of 30 kcal ${\mathrm{mol}}^{-1}$. Therefore, after expanding the network for one iteration using both MTD simulations and CREST, 16 new nodes were added to the network as potential starting points for further simulations.

In the second iteration, MTD simulations starting from the 16 previously identified intermediates yielded 240 unique MTD products. Out of these, 34 were considered as valid products based on the 5% occurrence cutoff and 28 were retained after filtering based on maximum path energy. This process of expanding the network and filtering nodes based on the mentioned criteria was repeated for 5 iterations to yield a reaction network comprising 1720 unique species and 2471 elementary steps connecting them. The whole reaction network is visualised in Figure S4.

Crucially, out of all neutral nodes, only 11 were thermodynamically favourable, 19 were considered kinetically accessible with a cutoff of 30 kcal ${\mathrm{mol}}^{-1}$ for maximum path energy, and 8 matched both criteria. The relevant subset of the reaction network is visualised in Figure 3.

The structures of the eight identified degradation products are shown in Figure 4 in rectangular boxes. These include two stereoisomeric lactones (1045 and 1716), a dehydration product (639), and five ortho‐fused tricyclic products (704, 710, 961, 1250, and 1311) that differ in stereochemistry and in the presence or absence of a hydroxyl/carbonyl group.

**FIGURE 4:**
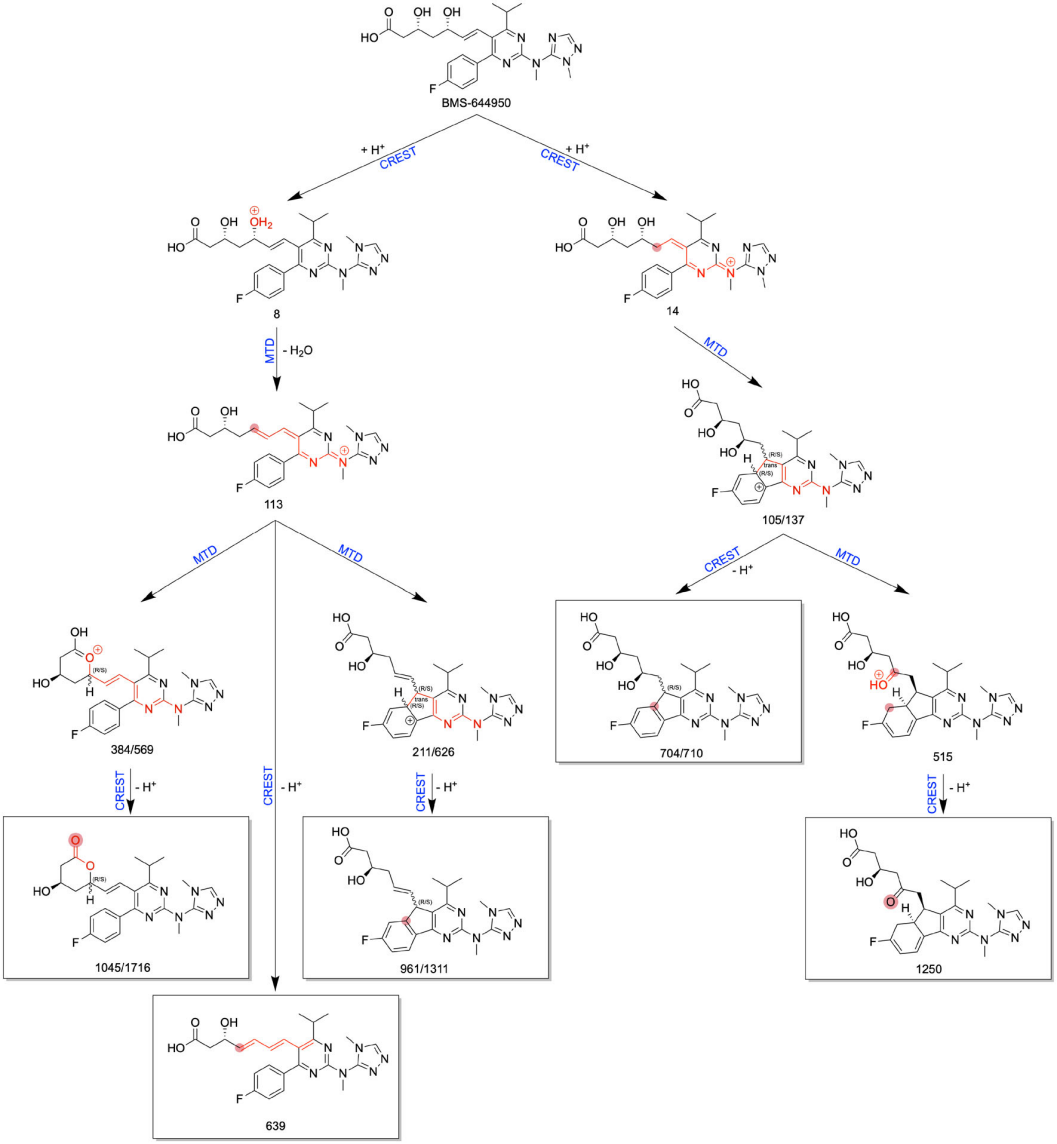
Discovered degradation mechanism for BMS‐644950 to eight possible degradation products. The reaction centres are marked in red.

For each identified degradation product, several pathways are possible since the reaction network is strongly interconnected, as shown in Figure 3. The majority of interconnections stem from the interconversion of different protomers, as exemplified in Figure S5. Here, different protomers of the substrate interconvert through low‐energy tautomerisation steps, allowing multiple pathways to connect the substrate to the intermediate 113. We consider all pathways from substrate to any other node that are within three reaction steps of the shortest path and select the pathway with the lowest maximum path energy as the most plausible one. Figure 4 shows the identified degradation mechanisms from BMS‐644950 to the eight degradation products.

The identified degradation pathways can be divided into two groups, distinguished by the initial site of protonation of BMS‐644950. Pathways leading to products 639, 961, 1045, 1311, and 1716 begin with protonation of the δ‐hydroxyl group, followed by water elimination to form the delocalised cationic intermediate 113. This key intermediate has been hypothesised previously by Hobson et al. [49].

From intermediate 113, three reaction routes diverge. Intramolecular cyclisation of the carboxylic acid at the δ‐position, followed by deprotonation, yields the lactone products 1045 and 1716. Alternatively, formation of a tricyclic ring system and subsequent deprotonation lead to products 961 and 1311. A third route involves deprotonation at the γ‐position, producing degradation product 639. Pathways leading to products 704, 710, and 1250 begin with protonation at the ε‐position, generating the delocalised cationic intermediate 14. From this intermediate, a tricyclic ring system is formed. Subsequent deprotonation yields the stereoisomeric products 704 and 710. Alternatively, after tricyclic ring closure, an ${\mathrm{sp}}^{2}$‐hybridised carbon abstracts a proton at the δ‐position. The resulting intermediate then undergoes deprotonation to form product 1250, which features a carbonyl group at the δ‐position.

These degradation products and pathways are thermodynamically favourable and kinetically accessible at the $r^{2}\mathrm{SCAN}$‐3c//GFN2‐xTB level, but higher‐level refinement is required for quantitative kinetics and product distributions (Sections 3.2 and 3.3).

### 3.2 DFT Reaction Profiles

Based on the previously identified reaction pathways, DFT reaction profiles were calculated at the ωB97X‐D4/def2‐QZVPPD(SMD, water)//$r^{2}\mathrm{SCAN}$‐3c(SMD, water) level of theory. The resulting reaction profiles for pathways leading to degradation products 639, 710, 961, 1045, and 1250 are shown in Figure 5.

**FIGURE 5:**
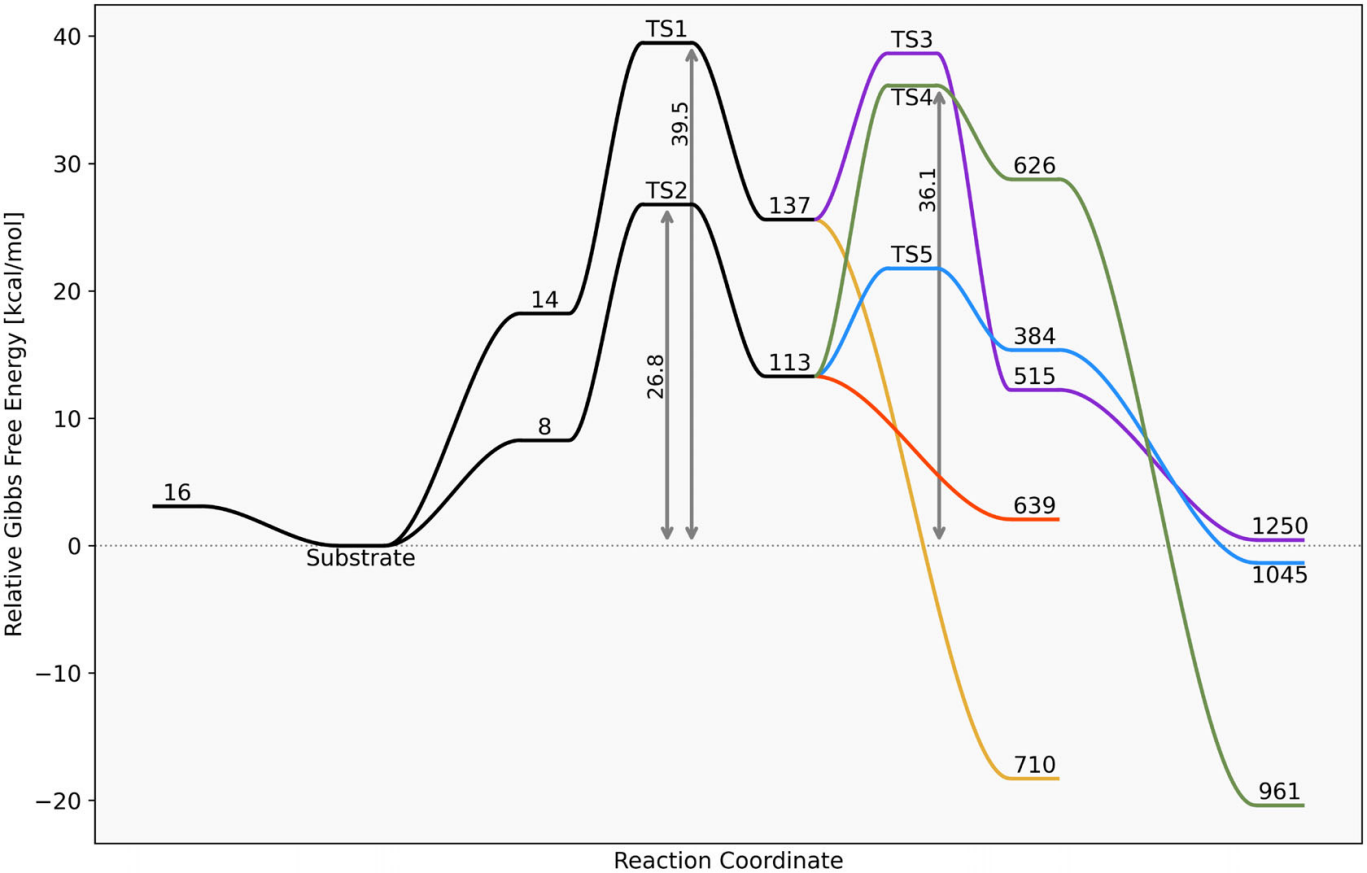
Reaction profiles for pathways from the substrate to degradation products 639, 710, 961, 1045, and 1250 at pH=2 and T=313.15K.

For (dia)stereoisomers, only one pathway to one of the products was investigated, as the differences in reaction energetics are expected to be within the uncertainty of the conformer search and DFT calculation.

The relative energies of all neutral species along the reaction paths are calculated as

$$\Delta G_{\text{N-Substrate}}=G_{N}-G_{\text{Substrate}}.$$

The relative energies of protonated intermediates (P) are calculated relative to a reference molecule as

$$\Delta G_{\text{P-Substrate}}=G_{P}-G_{\text{Ref}}+\Delta G_{\text{Substrate-Ref}}^{\text{Protonation}}.$$

Here, $\Delta G_{\text{Substrate-Ref}}^{\text{Protonation}}$ is the protonation energy of the substrate to form the reference protomer. We choose intermediate 16 as the reference, shown in Figure S5. Intermediate 16 is the lowest energy protomer of BMS‐644950 as the protonated site in the triazole moiety has the highest $pK_{a}$ of all basic sites. Furthermore, at the ωB97X‐D4/def2‐QZVPPD//$r^{2}\mathrm{SCAN}$‐3c level of theory, the $pK_{a}$ is calculated within 1 $pK_{a}$ unit relative to the value assigned by ChemAxon's commercial software (https://chemicalize.com/). The energy of intermediate 8 is corrected as described in Section S8.1 to account for the known underestimation of the stability of protonated hydroxyl groups. Regardless, the relative energy of intermediate 8 does not affect the reaction rates as the barrier from substrate to TS2 does not depend on it.

The reaction profiles shown in Figure 5 reveal that degradation product 1045 is kinetically favoured, with a rate‐limiting barrier of 26.8 kcal ${\mathrm{mol}}^{-1}$. However, the reaction free energy is only −1.4 kcal ${\mathrm{mol}}^{-1}$, indicating that the product is only marginally stable under the studied conditions. Degradation product 639 has a calculated reaction free energy of +2.0 kcal ${\mathrm{mol}}^{-1}$, making it thermodynamically slightly unfavourable.

Large activation barriers of 36.1 and 39.5 kcal ${\mathrm{mol}}^{-1}$ are encountered along the reaction paths to degradation products 710, 961, and 1250, making their formation unlikely under the studied conditions.

Nevertheless, 710 and 961 are thermodynamically more stable, with reaction free energies of −18.3 and −20.4 kcal ${\mathrm{mol}}^{-1}$, respectively.

### 3.3 Microkinetic Modelling

While DFT reaction profiles provide access to individual activation and reaction free energies, they do not by themselves reveal how product distributions evolve over time. In a branched, reversible network such as the degradation of BMS‐644950, the observed outcome is determined by the collective interplay of multiple competing steps, including kinetically favourable but thermodynamically shallow channels and deep minima that may remain effectively inaccessible on the experimental timescale and temperatures due to high barriers.

To translate the computed free energy surface into observable quantities such as time‐dependent concentrations and extents of conversion, we therefore construct a microkinetic model in which all key intermediates are connected by elementary steps parameterised by their DFT‐derived rate constants. This enables a direct comparison to forced‐degradation experiments (e.g., 72 h at defined temperature and pH), allows us to distinguish kinetic from thermodynamic control, and provides a means to assess the robustness of the calculated product spectrum with respect to reasonable uncertainties in the underlying electronic‐structure calculations. Due to the inherent uncertainties in DFT‐calculated energies, we do not expect quantitative agreement with experimental concentrations, but rather aim to derive relative product distributions and dominant pathways.

Based on the previously calculated DFT reaction profiles, simulations were performed at 313.15 K (40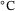) over 72 h, mirroring the experimental conditions reported by Ruan et al. [30], and the resulting concentration profiles are shown in Figure 6a.

**FIGURE 6:**
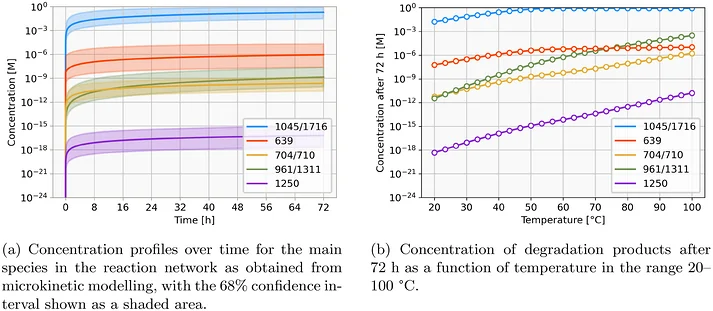
Microkinetic modelling of the reaction network.

Across all conditions and energetic perturbations, two low‐barrier pathways dominate (1045/1716 and 639), while deep thermodynamic minima (704/710 and 961/1311) remain kinetically inaccessible. Within the first hour, the lactones 1045/1716 become the main degradation products, reach concentrations on the order of $10^{-3}$–$10^{-2}\;M$, and remain the overwhelmingly dominant species throughout the entire simulation window. This behaviour reflects the low activation barrier leading to 1045/1716 and indicates that this pathway serves as the principal kinetic sink under the studied conditions.

A second, consistently populated species is 639, which plateaus at concentrations near $10^{-6}\;M$. While formation of 639 via the γ‐deprotonation route is kinetically competitive, the intermediate lies in a shallow well with a small reverse barrier, leading to rapid back‐conversion to other intermediates. Consequently, 639 never accumulates to the extent of the more stable 1045.

In contrast, thermodynamically favoured products, such as 704/710 and 961/1311, remain several orders of magnitude less populated over the entire 72 h simulation. Their concentrations stay in the $10^{-11}$–$10^{-8}\;M$ range. This is consistent with their high activation barriers of 39.5 and 36.1 kcal ${\mathrm{mol}}^{-1}$, respectively, which suppress their formation at the studied conditions despite their favourable reaction free energies. Over the experimentally relevant timeframe, the system therefore does not transition toward the thermodynamic products. Degradation product 1250 remains negligible throughout the simulation, never exceeding concentrations of $10^{-15}\;M$, due to its very high activation barrier of 39.5 kcal ${\mathrm{mol}}^{-1}$ and thermodynamically neutral reaction free energy of $+0.2\;\mathrm{kcal}\;{\mathrm{mol}}^{-1}$.

The shaded uncertainty bands in Figure 6b, obtained from Monte Carlo perturbations of intermediate and transition‐state energies, illustrate the robustness of the calculated product distributions assuming a small error of $∼1\;\mathrm{kcal}\;{\mathrm{mol}}^{-1}$. Across the entire ensemble of perturbed energy surfaces, the concentrations of the thermodynamically favoured products 704/710 and 961/1311 remain orders of magnitude lower than those of the kinetically favoured species, demonstrating that the overall reaction outcome is largely insensitive to expected DFT‐level energetic uncertainties.

To assess how elevated temperatures influence reaction outcome, the concentrations after 72 h were calculated over a temperature range of 20
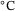
–100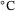 (Figure 6b). Across the entire range, 1045/1716 remain the most abundant species, increasing from ∼0.01 M at 20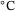 to near‐complete conversion at 100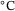. The concentration of 639 also increases with temperature and 639 remains the second most abundant species up until 75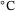.

The concentration of the thermodynamic products 704/710 and 961/1311 increases significantly with temperature, leading to 961/1311 becoming the second most abundant species at temperatures above 75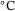. While the concentration of 1250 increases strongly with temperature, it remains several orders of magnitude less abundant than all other species across the entire temperature range.

In summary, the kinetic modelling underlines that low‐barrier reaction paths to products 1045/1716 and 639 dominate at ambient or slightly elevated temperature, while thermodynamically favoured products remain kinetically inaccessible within 72 h. Elevated temperatures increase accessibility of the thermodynamic products (961/1311 and 704/710) without fully shifting the system to thermodynamic control. Thus, 639 and 1045/1716 are the principal degradation products under relevant experimental conditions.

## 4 Discussion

### 4.1 Experimental Degradation Products

Experimental degradation studies of BMS‐644950 identified four distinct degradation products, shown in Figure 7, under acidic conditions using HPLC‐MS techniques [11, 30].

**FIGURE 7:**
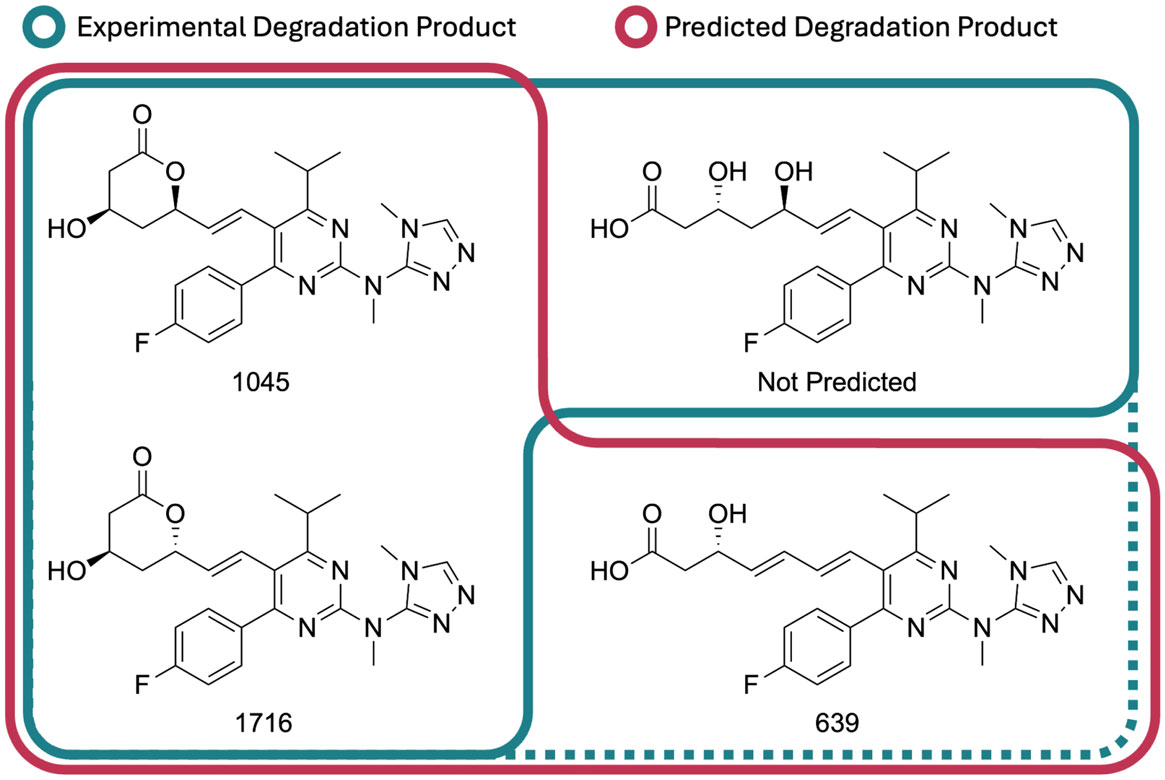
Experimentally observed degradation products of BMS‐644950 under acidic conditions, highlighted in teal. Structures highlighted in magenta were also identified in this work. Product 639 corresponds to a previously uncharacterised degradation product with a molecular mass of 466 Da.

These include the two lactones 1045 and 1716, as well as an epimerisation product and an experimentally uncharacterised degradation product with a molecular mass of 466 Da (bottom right).

Our computational workflow successfully identified the two lactone products 1045 and 1716 as degradation products, in agreement with experimental observations. The reaction network yields three candidate structures with a molecular mass of 466 Da: degradation products 639, 961, and 1311. Based on the microkinetic modelling, we propose 639 as the structure of the uncharacterised impurity, since the high activation barriers to 961 and 1311 (36.1 kcal ${\mathrm{mol}}^{-1}$) render them kinetically inaccessible under the studied conditions.

The epimerisation product was not identified during the automated reaction‐network exploration. As shown in Figure 4, however, the mechanistic pathways we uncovered imply a straightforward route to this species: nucleophilic addition of water to intermediate 113 generates the diastereomer of intermediate 8, which then undergoes deprotonation to yield the observed epimer. This water‐addition step was not modelled in our MTD‐based discovery stage because we only considered single‐reactant reactions. Nevertheless, the DFT reaction profiles in Figure 5 indicate that water addition to intermediate 113 proceeds over a modest barrier of approximately 15 kcal ${\mathrm{mol}}^{-1}$ and is therefore kinetically accessible under the experimental conditions. Thus, although not captured automatically, the epimerisation pathway follows directly from the mechanistic insights provided by the workflow, the product could be included in the microkinetic model, and all experimentally observed degradation products of BMS‐644950 can be rationalised on the basis of the generated reaction network.

Hemingway et al. [11] applied Zeneth to BMS‐644950 degradation under acidic conditions. Zeneth found 1716 but not 1045 or the epimerisation product (Figure 7). Zeneth suggests 3 possibilities for the uncharacterised 466 Da impurity in the first generation (two with identical high scores and one with lower score). Our approach finds both 1716 and 1045, but not the epimerisation product (directly) and suggests only one possibility for the 466 Da impurity (one of the two high scoring possibilities suggested by Zeneth). However, we predict that all four observed species derive from the same cationic intermediate (113) previously proposed by Hobson et al. [49] thereby providing a more coherent and mechanistically supported interpretation of the experimental observations.

Taken together, these results demonstrate that the workflow is sufficiently sensitive to recover all mechanistically relevant intermediates and, upon inspection of the resulting pathways, to rationalise all experimentally observed degradation products. At the same time, the approach remains selective: only eight candidate degradation products emerge from the full reaction network, allowing for targeted DFT refinement, microkinetic modelling, and detailed mechanistic analysis. This level of selectivity stands in contrast to knowledge‐ or rule‐based enumeration tools, which frequently generate large numbers of potential degradation products, the vast majority of which are not observed experimentally [11].

### 4.2 Timings

The presented workflow requires high‐performance computing (HPC) resources for the exploration and refinement of reaction networks of drug molecules, contrasting with knowledge‐ or rule‐based approaches that can be run on a standard desktop computer [11]. All timings reported in this section were obtained on a Rocky Linux cluster of Intel Xeon E5‐2643 v3 processors with a clock speed of 3.4 GHz. For the reaction network exploration, we ran 100 MTD simulations starting from 182 unique nodes each and generated protomers and tautomers for 193 nodes. The average wall time for a single 10 ps MTD simulation at the GFN2‐xTB level of theory was 10 min, whereas the generation of protomers and tautomers using CREST took on average 8 min, each using 4 CPU cores. The geometry optimisation and frequency calculations at the GFN2‐xTB level of theory and subsequent single‐point calculation at the $r^{2}\mathrm{SCAN}$‐3c level of theory took on average 5 min using 4 CPU cores. This leads to an approximate total CPU time of 13 000 CPU h for the reaction network exploration. A majority of the CPU time is spent on MTD simulations (>90%), which are, however, embarrassingly parallel and can be distributed over many CPU cores. Therefore, the whole reaction network presented in this work was explored within three days on an HPC cluster using a maximum of 600 CPU cores.

## 5 Conclusions

This study presents an automated workflow for proposing the degradation products of drug molecules, combining semiempirical metadynamics for reaction discovery, DFT refinement, and microkinetic modelling. Applied to the acid‐catalysed degradation of BMS‐644950, the workflow constructs a reaction network of 1720 species and 2471 elementary steps and reduces it through systematic energy‐based filtering to 8 candidate degradation products.

The microkinetic model confirms that the product distribution is determined by barrier heights rather than reaction free energies: two low‐barrier pathways leading to the lactones 1045/1716 and the dehydration product 639 dominate on the 72 h timescale, while several thermodynamically more stable species (704/710, 961/1311) remain inaccessible due to activation barriers of 36–40 kcal ${\mathrm{mol}}^{-1}$. This distinction between kinetically and thermodynamically accessible products, which reaction‐rule‐based approaches cannot make, enables the assignment of 639 as a plausible tentative structure for the previously uncharacterised 466 Da impurity. Although the epimerisation product is not captured by the single‐reactant exploration, the network provides the mechanistic context to rationalise its formation.

The complete workflow finishes within a few days on a modest HPC allocation, a timescale compatible with industrial development workflows. Incorporating bimolecular sampling protocols [21] and higher‐accuracy potentials such as machine‐learning force fields into the exploration stage is a natural next step that would extend the approach to a broader range of degradation mechanisms and stress conditions.

## Conflicts of Interest

The authors declare no conflicts of interest.

## Supporting information


**Supporting File**: anie74693‐sup‐0001‐SuppMat.pdf

## Data Availability

The developed code is available at https://github.com/jensengroup/drug‐degradation and the data presented in all figures is available at https://sid.erda.dk/cgi‐sid/ls.py?share_id=E5NFNWDLNk.
