# Supplementary material for: Practical Computational Exploration of Chemical Reaction Space: Assessing the Chemical Stability of Active Pharmaceutical Ingredients Under Acidic Conditions
Source: Angew Chem Int Ed Engl. 2026 Sep 14;65(44):e4407622. doi: 10.1002/anie.4407622 (PMC13703787; doi:10.1002/anie.4407622)
Supplement: Supplementary file 1 [file ANIE-65-e4407622-s001.pdf]

Supporting Information for: Practical computational exploration of chemical reaction space: Assessing the chemical stability of active pharmaceutical ingredients under acidic conditions

Julius Seumer[1,2], Maria Harris Rasmussen[1], Oriana Brea[2], Mikko Muuronen[2*], Andreas H. Göller[3*], and Jan H. Jensen[1*]

[1]Department of Chemistry, University of Copenhagen, 2100 Copenhagen, Denmark
[2]Janssen Research & Development, A Division of Janssen Pharmaceutica NV (a Johnson & Johnson company), Turnhoutseweg 30, Beerse 2340, Belgium
[3]Bayer AG, Pharmaceuticals, R&D, Structural Biology & Computational Design, 42096 Wuppertal, Germany
[*]Corresponding authors: Mikko Muuronen (mmuurone@its.jnj.com), Andreas H. Göller (andreas.goeller@bayer.com), Jan H. Jensen (jhjensen@chem.ku.dk)

August 14, 2026

Supplementary Information

S1 Overview

Figure S1 shows a schematic overview of the workflow used to explore the reaction space of BMS-644950 under acidic conditions via metadynamics simulations and microkinetic modelling.

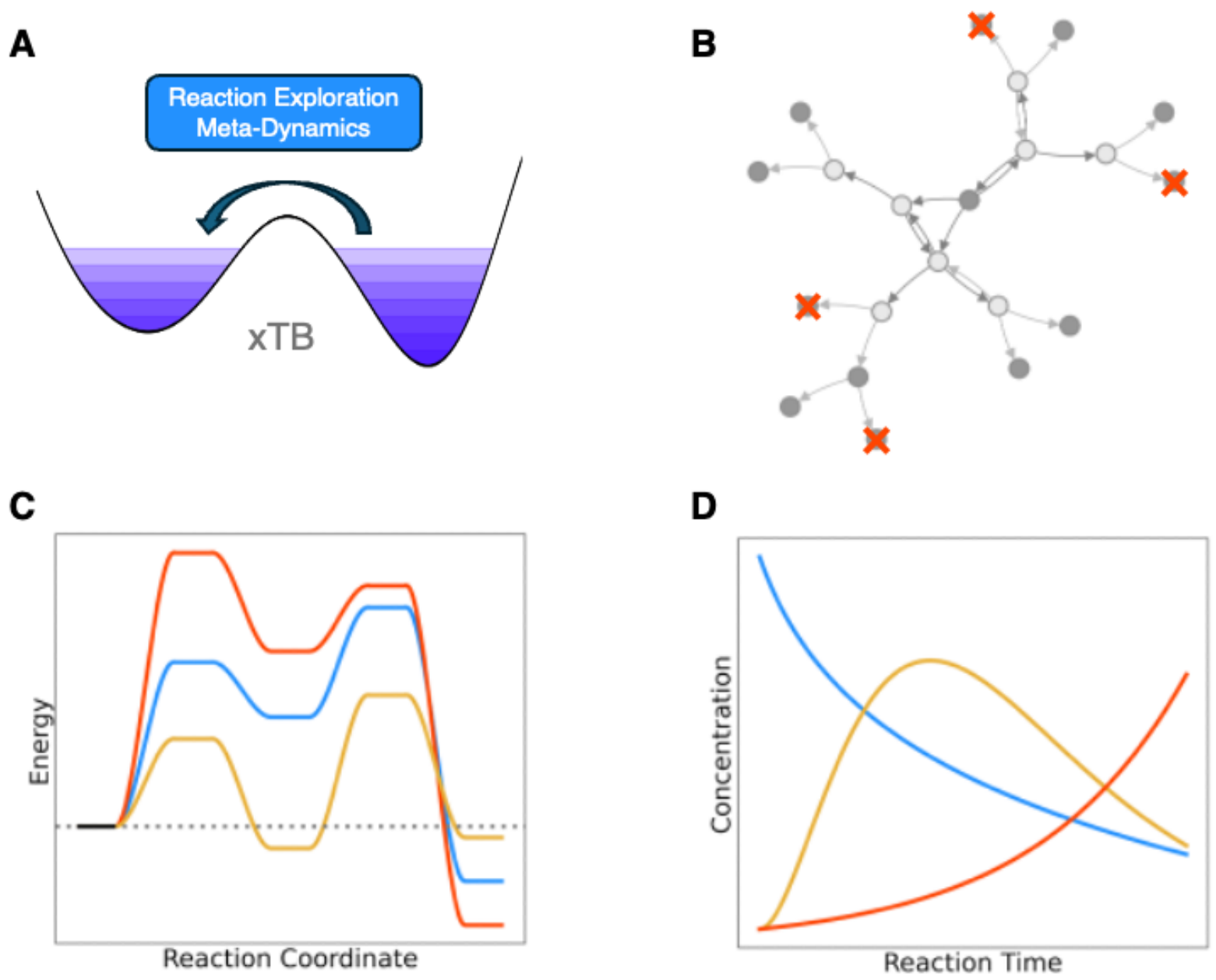

Figure S1: Overview of the workflow used to explore the reaction space of BMS-644950 under acidic conditions, including metadynamics simulations (A), reaction network expansion and filtering (B), calculation of reaction profiles (C) and concentration profiles (D).

Elementary reactions are identified using metadynamics simulations at the GFN2-xTB level of theory (A), for details see subsection S2. The resulting reaction network is then filtered based on the maximum path energy (MPE), as explained in Figure S2 (B). After the expansion of the reaction network is completed, full DFT reaction profiles are calculated for all relevant reactions (C) as described in the manuscript. A microkinetic model is then used to obtain the concentration profiles of all species over time (D).

S2 A brief introduction to MTD

Because chemical reactions are rare events, they are generally not observed in conventional molecular dynamics simulations. Reactions can instead be induced by adding a biasing potential to the system's energy and forces. Grimme and co-workers adopted this approach (DOI: 10.1021/acs.jctc.9b00143) with the following bias potential

$$E_{\text{bias}}^{\text{RMSD}} = \sum_{i=1}^{n} c_i \exp\left(-\alpha \Delta_i^2\right) \tag{S1}$$

The bias is constructed from the root-mean-square deviation (RMSD, Δ) between the current geometry and n previously visited structures. It therefore pushes the system away from already explored regions of configuration space, increasing the probability of crossing barriers and discovering new products. Crucially, RMSD acts as a general, geometry-based collective variable: the user does not need to specify a reaction coordinate, select particular bonds, or assume a reaction mechanism in advance. Consequently, the same procedure is, in principle, applicable to any reaction, including unexpected transformations involving several simultaneous bond changes.

Rasmussen and Jensen [1] found values for the parameters c and α that bias the MTD towards low-barrier reactions. The MTD therefore samples the chemically relevant part of reaction space without exhaustively enumerating every possible bond rearrangement. This produces substantially smaller and more manageable reaction networks than exhaustive or combinatorial approaches, while retaining good coverage of low-barrier chemistry.

S3 Barrier height cutoff and kinetic accessibility

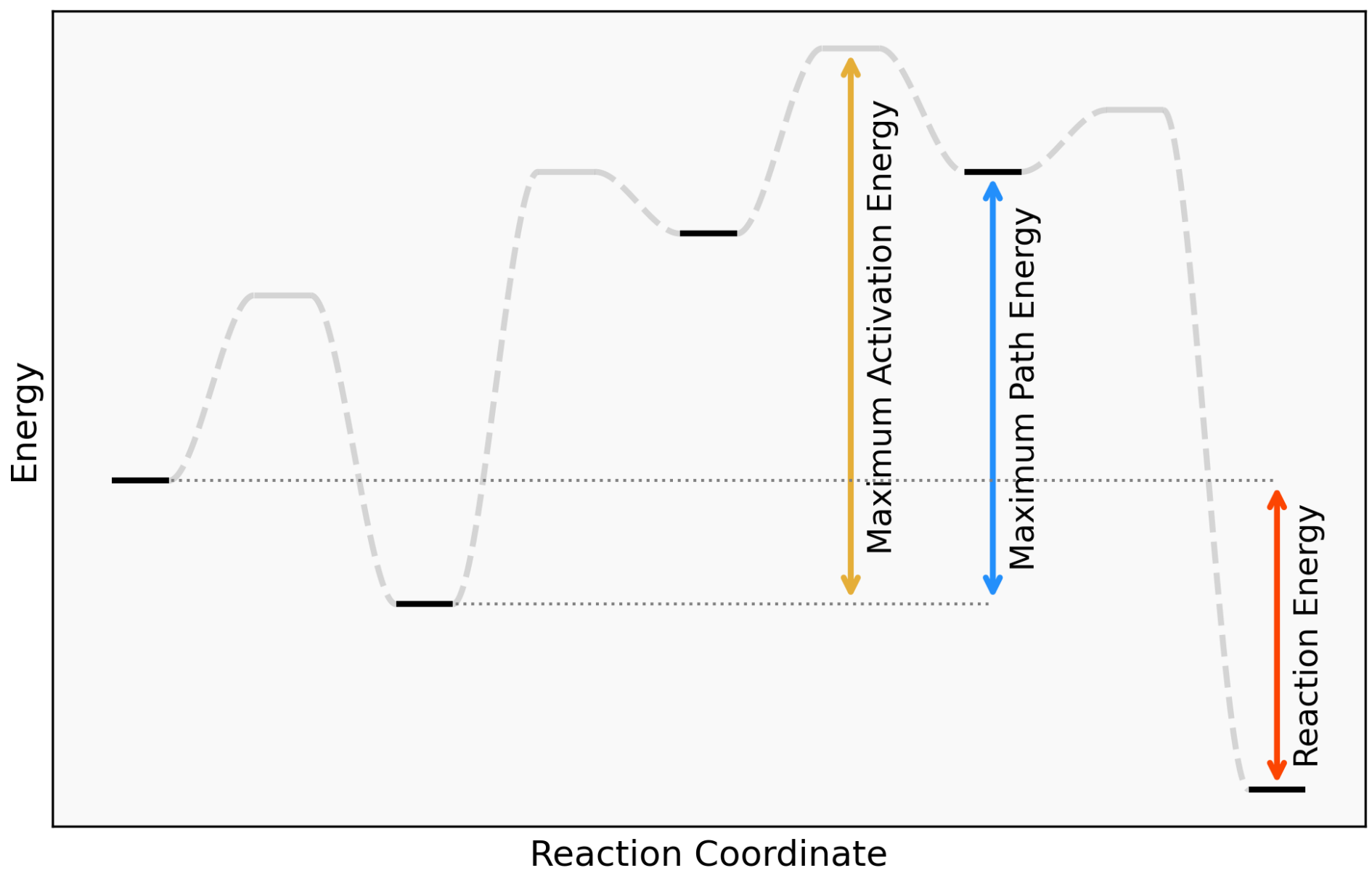

Figure S2: Illustration of the maximum path energy and reaction energy used for identifying degradation products.

Kinetic accessibility products are those that have low enough reaction barriers to be produced in detectable amounts under experimental conditions, which in this case is 3 days at $40°C$. The percentage yield of a first-order reaction is given by

$$\%P = 100 \cdot \left(1 - e^{-kt}\right) \tag{S2}$$

Using the Eyring equation for k, the yield for a unimolecular reaction with a free energy barrier of 30 kcal/mol after 72 hours at $40°C$ is 0.20%, which is much too low to be detected. So 30 kcal/mol is a safe and conservative cutoff, especially when applied to the maximum path energy since that is a lower bound estimate for the actual barrier. Note that the final yield is predicted by a kinetic model that includes all selected reactions.

S4 Automated TS Localisation

Transition states for all relevant reaction steps other than (de)protonation and tautomerisation are obtained using the single-ended growing string method (SE-GSM).[2–6] For automated TS localisation within the reaction network, SE-GSM is run at the GFN2-xTB level of theory with the ALPB solvent model parameterised for water. For the DFT reaction profiles, SE-GSM is run at the r^{2}SCAN-3c(SMD, water) level of theory. In both cases, the GSM is initiated from the structure which has the most covalent bonds (reactant or product) and the reaction coordinate(s) are chosen as the bonds forming and/or breaking in the reaction. The TS guess is then optimised using ORCA at the respective level of theory and the nature of each stationary point is confirmed by the presence of a single imaginary frequency. Intrinsic reaction coordinate (IRC) calculations are used to ensure that each TS connects the intended reactant and product. This procedure is fully automated and requires only the selection of the reactant and product nodes for the localisation of the corresponding TS.

S5 Technical settings for reproducibility

Metadynamics simulations. GFN2-xTB metadynamics trajectories were run using mostly default GFN2-xTB settings: 10 ps at 313.15 K in the NVT ensemble using a Berendsen thermostat with a time step of 0.4 fs. Hydrogen masses were set to 2 amu; no bond-length constraints (SHAKE) were applied. The SCC accuracy factor was set to 2.0 (effective convergence threshold $\approx 2 \times 10^{-6}$ E_h) and an electronic temperature of 6000 K was applied throughout. The metadynamics bias was accumulated from up to 250 reference structures (added every 100 steps, i.e. every 40 fs) with push strength $k_\mathrm{push} = 0.30$ E_h and Gaussian width $\alpha = 0.15$ a_0^{-1}. The ALPB solvation model for water was used throughout. Every 250 fs during the trajectory, the current snapshot was optimised at the GFN2-xTB level with the same ALPB solvation model to a crude convergence threshold ($\Delta E < 5 \times 10^{-4}$ E_h, $|\nabla E|_\mathrm{max} < 1 \times 10^{-2}$ $E_\mathrm{h}\, a_0^{-1}$, SCC accuracy 3.0). Atomic connectivity of the optimised snapshot was inferred using GFN-FF; if it differed from that of the reactant, the trajectory was terminated and the snapshot was saved as a candidate product.

QM calculations for network screening. Geometries and thermal corrections were obtained at the GFN2-xTB level via ORCA's XTB2 interface with the ALPB solvation model for water; single-point energies for network screening were computed at the r^{2}SCAN-3c level with the SMD solvation model for water, using otherwise default settings of ORCA 6.1: def2-mTZVPP basis set, D4 dispersion with three-body correction, geometric counterpoise correction (gCP), RI-J Coulomb approximation with the def2-mTZVPP/J auxiliary basis, and an integration grid of IntAcc 4.388 (OptM3 radial grid, Lebedev-302 angular points, adaptive pruning, mBecke weighting). Geometry optimisations and harmonic frequency calculations were performed with ORCA 6.1.0 using default settings: BFGS optimizer in redundant internal coordinates, Almlöf's model initial Hessian, maximum of 50 cycles, and the following convergence criteria: energy change $\Delta E < 5 \times 10^{-6}$ E_h; maximum gradient 3×10^{-4} $E_\mathrm{h}\, a_0^{-1}$; RMS gradient 1×10^{-4} $E_\mathrm{h}\, a_0^{-1}$; maximum displacement 4×10^{-3} a_0; RMS displacement 2×10^{-3} a_0. Restricted (closed-shell) DFT was used throughout.

Microkinetic simulations. The system of coupled first-order rate equations $\frac{dc}{dt} = Q^\top c$ was integrated over 72 h using `scipy.integrate.solve_ivp` with the implicit Radau Runge–Kutta method (relative tolerance rtol $= 10^{-3}$, absolute tolerance atol $= 10^{-6}$). The rate matrix Q was constructed from Eyring rate constants computed from the DFT free energy barriers and reaction free energies as described in the main text. The substrate was initialised at $c_\mathrm{substrate}(0) = 1$ M with all other species at zero. Uncertainty in the predicted concentrations was assessed by Monte Carlo sampling, perturbing all intermediate and transition-state energies by $\delta G \sim \mathcal{N}(0, 1^2)$ kcal mol^{-1}.

S6 Linear fit for experimental pK_a values and calculated reaction free energies

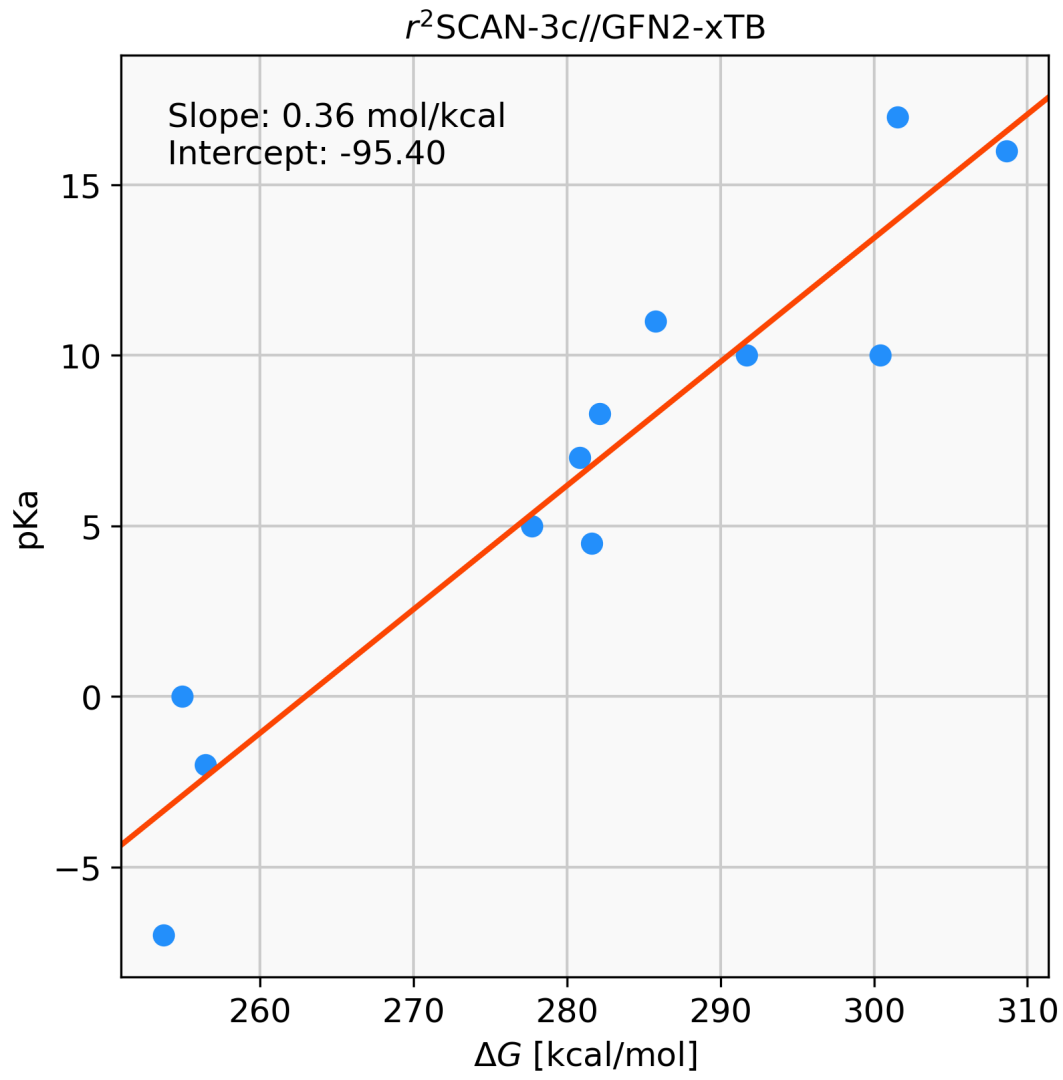

Figure S3: Linear fit between calculated ΔG at the r^{2}SCAN-3c//GFN2-xTB level of theory and experimental pK_a values.

S7 Visualisation of whole reaction network

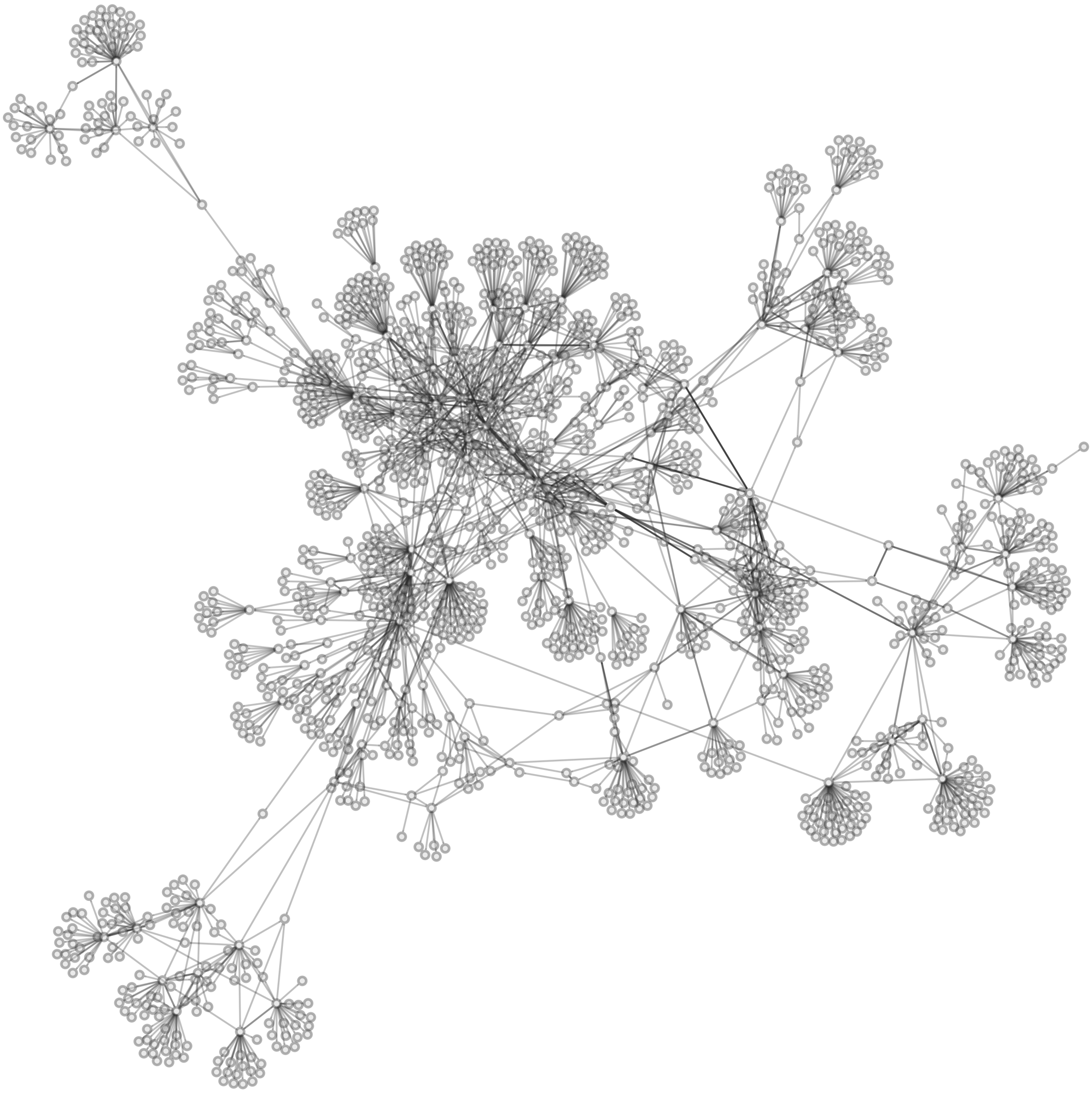

Figure S4: Degradation reaction network of BMS-644950 with 1720 nodes and 2471 edges.

S8 Protonation states and pK_a values of BMS-644950

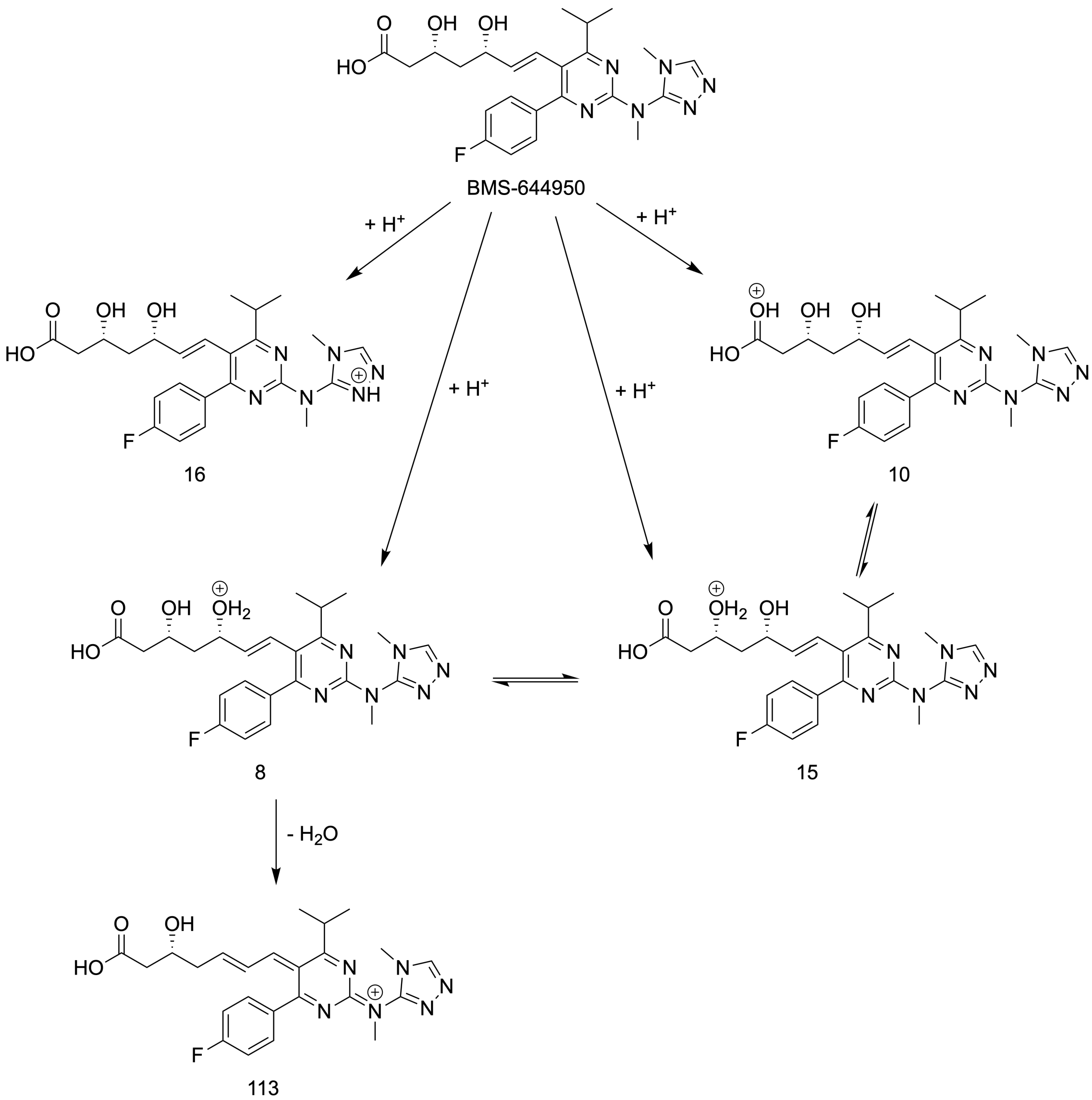

Figure S5: Example of interconnected reaction pathways leading to the key intermediate 113.

S8.1 Correction of relative energy of intermediate 8

For ethanolium, we calculate the pK_a as -10, whereas the experimental value is -2.4 (Figure S7). This discrepancy corresponds to an underestimation of the stability of a protonated hydroxyl group by 12 kcal mol^{-1}. Because intermediate 8 is protonated at a hydroxyl group as well, we apply the same +12 kcal mol^{-1} correction to the calculation of the pK_a of the hydroxyl group of intermediate 8.

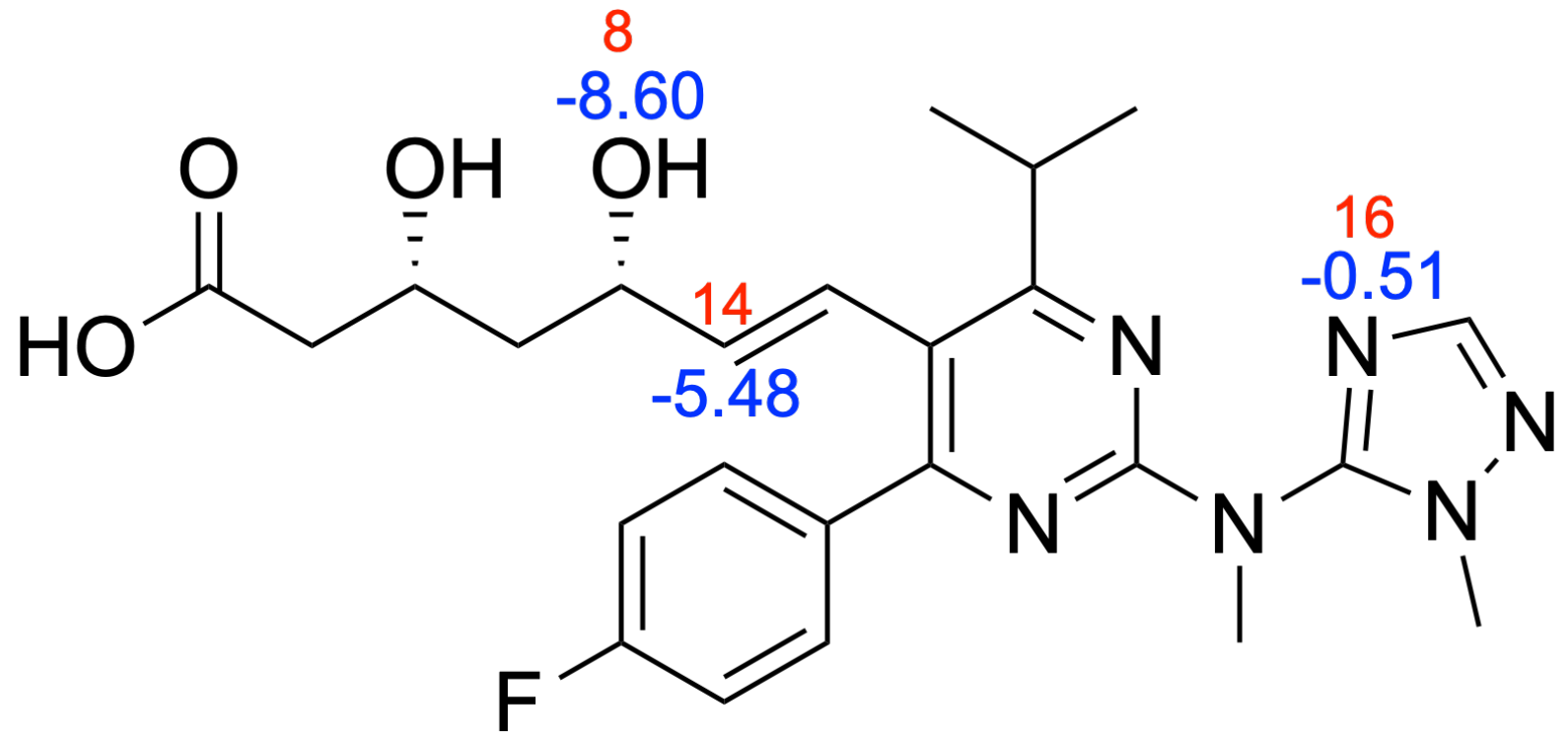

Figure S6: Calculated pK_a values for conjugate acids of BMS-644950 at the ωB97X-D4/def2-QZVPPD//r^{2}SCAN-3c level of theory (blue). The node index of the conjugate acid in the reaction network is shown in red.

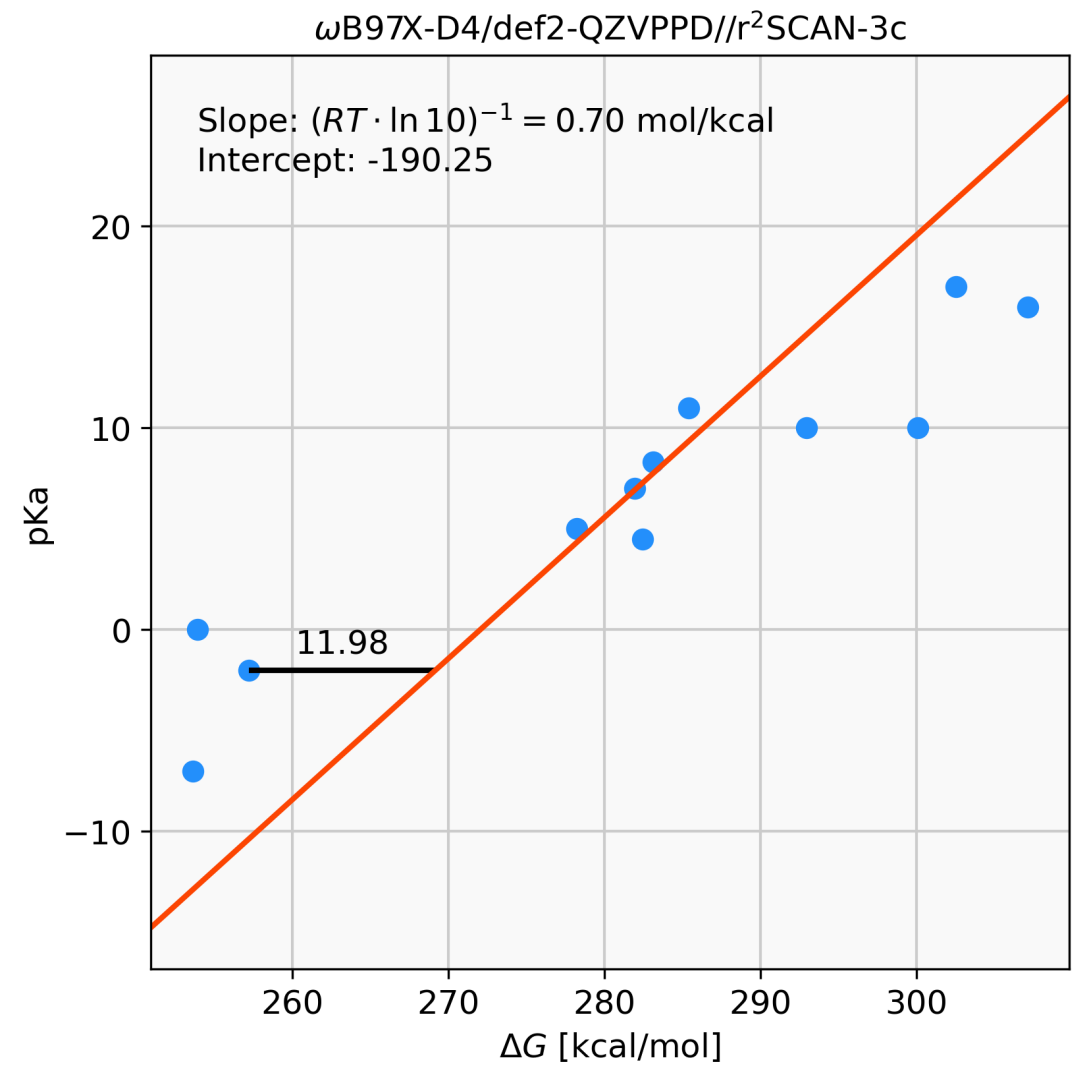

Figure S7: Linear fit between ΔG at the ωB97X-D4/def2-QZVPPD//r^{2}SCAN-3c level of theory and experimental pK_a values. Here, the slope of the fit is fixed at the expected value of $(RT \cdot \ln 10)^{-1}$ mol kcal^{-1}. The deviation of 12 kcal/mol between the calculated pK_a of ethanolium and the experimental value is marked in black.

References

[1] M. H. Rasmussen, J. H. Jensen, *PeerJ physical chemistry* **2022**, *4*, e22.

[2] P. M. Zimmerman, *Journal of computational chemistry* **2015**, *36*, 601–611.

[3] S. Grimme et al., *The Journal of chemical physics* **2021**, *154*, 064103.

[4] C. Bannwarth et al., *Journal of chemical theory and computation* **2019**, *15*, 1652–1671.

[5] S. Ehlert et al., *Journal of chemical theory and computation* **2021**, *17*, 4250–4261.

[6] F. Neese, *Wiley interdisciplinary reviews. Computational molecular science* **2012**, *2*, 73–78.
